# Ageism in an Aging Society: The Role of Knowledge, Anxiety about Aging, and Stereotypes in Young People and Adults

**DOI:** 10.3390/ijerph16081329

**Published:** 2019-04-13

**Authors:** Anna Rosa Donizzetti

**Affiliations:** Department of Humanities, University of Naples Federico II, 80133 Napoli NA, Italy; donizzet@unina.it

**Keywords:** ageism, stereotypes, anxiety about aging, knowledge, chronological age, structural equation modeling

## Abstract

The progressive aging of society, caused by profound demographic changes, brings with it the necessity of confronting the subject of biases against the elderly. Ageism, in fact, can influence society’s attitudes regarding this population, in addition to impacting the self-perception of elderly people. This, in turn, has consequences for positive outcomes during the aging process. The current research aims to investigate the simultaneous relationships between knowledge, age, anxiety about aging, and stereotypes toward the elderly, as well as their predictive roles with respect to ageism. A self-report questionnaire was administered to 886 participants, with an average age of 35.8 years (Standard Deviation—SD = 14.2), predominantly female (64.8%). Descriptive and correlational analyses were performed, along with structural equation modeling. Based on the analyses conducted, anxiety about aging and knowledge are antecedents for stereotypes, which in turn, together with the other variables, influence ageism. Increased education about the aging process could help reduce anxiety and stereotypes against the aging among those who are most responsible for prejudice against the elderly. Knowledge of the antecedents of prejudice toward the elderly is fundamental to promoting positive attitudes toward them.

## 1. Introduction

Demographic changes, due to longer average lifespans and lower birth rates, are impacting all nations in different ways. The effect of these changes is a large increase in the elderly population, which forces us to consider aging as a social problem with global impact. As a result, countries find themselves confronted with the need to reorganize themselves in order to address the socio-economic impacts of aging populations. Reorganization means not only finding the appropriate tools to help the oldest among us, but also creating ways to involve and value the resources and potential of the youngest segments of the elderly population. In order for this process to be successful, one must take stock of the psycho-social impact of this demographic shift and subsequent reorganization. In fact, over the years, rising prejudices have spread concerning the elderly, who are seen as hindering productivity and social dynamism [1]. Stereotypes about aging, beyond influencing behavior and ways of managing the care of elderly populations, can also impact personal experiences of aging. Negative self-perception of aging involves reduced self-efficacy, with direct effects on depression [2], along with repercussions for physical health, due to effects on the immune system [3] and on the cardiovascular system [4]. Meanwhile, positive self-perceptions of aging are associated with higher levels of well-being, better health, and/or longevity [5,6]. Therefore, the goal is to contrast stereotypical visions of aging with new approaches, like that of positive aging [7] and successful aging [8], which conceptualize existence no longer as directed toward inevitable decline [9], but rather as a process shaped by the individuals and the context in which they live [7]. Until we bring about this shift in perspective, however, it is necessary to unpack prejudices regarding the elderly. These biases can discourage elderly people from freely participating in work or recreational activities and, furthermore, they can contribute to the social isolation of the oldest generations, limiting their ability to make a positive contribution to the collective whole, and perpetuating fear of aging in all individuals [10]. To this end, this contribution seeks to expand our knowledge of the antecedents for prejudice against the elderly, with the goal of implementing programs that promote more positive attitudes toward them.

### 1.1. Research Framework

Butler [11] was the first to use the term ‘ageism’ to describe prejudice against the elderly, defining it as “a process of systematic stereotyping of and discrimination against people because they are old” (p. 12). In the over forty years that have passed since this definition was coined, various others have been proposed, which have attempted to capture the complexity of this phenomenon and its diversity with respect to other, more well-known forms of prejudice. The most complete definition has been offered by Iversen, Larsen, and Solem [12], who, after a review and analysis of all the definitions given over the years, defined ageism as “negative or positive stereotypes, prejudice and/or discrimination against (or to the advantage of) elderly people on the basis of their chronological age or on the basis of a perception of them as being ‘old’ or ‘elderly’. Ageism can be implicit or explicit and can be expressed on a micro, meso, or macrolevel” (p. 15). This definition is particularly interesting because, beyond emphasizing aspects already well-recognized in the literature, such as the classic social-psychological components (cognitive, affective, and behavioral) and the conscious and unconscious dimensions, it underlines the individual, social, and institutional significance of the phenomenon. Indeed, the studies found in the literature can be divided into three major categories: the first, which will concern us the most, focuses on individual aspects [13]; the second has examined social factors such as isolation [14]; and the third category looks at institutional elements, such as being fired from work, limited employment opportunities in the job market, and career choices [15,16,17].

According to Levy’s stereotype embodiment theory [18], the process of internalizing the age stereotypes that permeate society begins during childhood and continues afterwards [19]. Indeed, the presence of ageism has been observed in all age ranges, from small children through to the oldest populations [20,21], so much so that the elderly themselves describe having experienced some form of prejudice at least once during their lives [22]. Even if the studies conducted on this subject have led to differing and occasionally contradictory results [23], much research has documented the existence of negative stereotypes toward the oldest people, not only in the United States [22,24], but also in other parts of the world, like the Far East (China, Japan, Thailand) [25] and the Middle East (for example, Turkey) [26]. In Italy [27], there has been a greater prevalence of stereotypes, rather than prejudice, toward the elderly. The spread of stereotypes has been attributed to a lack of knowledge about the aging process, while the relative absence of discriminatory tendencies has been interpreted in terms of cultural influences [25]. In fact, in Italy, as in other collectivist cultures [28], there are robust values that support the elderly, which put a strong emphasis on affective ties among family members. Studies have shown, furthermore, that males and young people demonstrate the highest levels of ageism, relative to women and the elderly [27,29,30,31]. Contradictory results have been found with respect to the correlation between ageism and age. Some researchers [30,31] have found a significant negative correlation, while Hellbusch and colleagues [32] found that elderly people are more prejudiced against their own age group, relative to younger age groups. Other researchers were unable to find any kind of correlation at all [33].

There have been numerous other studies conducted in order to understand the antecedents of prejudice toward the elderly. First among these, the role of knowledge about aging has been investigated [34]. It has been shown that knowledge is intrinsically related to attitudes, and the acquisition of new knowledge concerning a given object or population has been considered one of the most effective methods of changing attitudes [35]. The level of knowledge, among those with high levels of prejudice, is generally low [36]. Many other studies have shown the existence of a negative correlation, statistically significant, between knowledge about aging and stereotypes toward the elderly [37,38,39,40]. At the same time, however, other studies were unable to find a significant relationship between the two constructs [41]. The contradictory nature of these results has been attributed to the changes made to the survey instruments and to problems related to sampling [42]. Knowledge about aging has been negatively correlated with ageism [42,43,44], even if, for university students, a greater knowledge of the process of aging translates to a reduction in the level of ageism only indirectly, via the mediation of anxiety about aging [37]. Furthermore, knowledge about aging is negatively correlated with anxiety about aging [41,45,46]. Anxiety about aging can be defined as “the combined concern and anticipation of losses centered around one’s own aging process” [47] (p. 247). Referring to the personal fears of individuals with respect to the changes associated with aging, anxiety is relative to ‘me’, which is different from ageism which, referring to attitudes toward members of an outgroup based on age, is relative to ‘them’. Therefore, we are dealing with two different constructs which are positively correlated between themselves [13,42,48]. Anxiety about aging is also positively correlated with stereotypes toward the elderly [41], and negatively correlated with age [47].

### 1.2. The Current Research

In light of the various results of research on the topic, the aim of the current study was to investigate the relationships between knowledge about aging, age, stereotypes about the elderly, anxiety about aging, and ageism. Analysis of the simultaneous relationships between these variables can provide a more complete vision, given the existing fragmentary studies on the subject.

**Figure 1:**
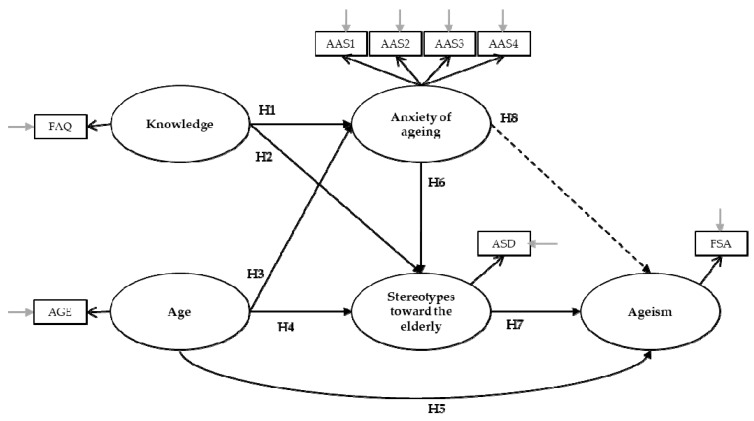
Conceptual model.

Based on an extensive review of the literature, we constructed an a priori model to test. The model represented the known relationships between the variables of interest and ageism. As shown in Figure 1, we expected that knowledge would negatively predict anxiety about aging (H1) and stereotypes toward the elderly (H2). We expected that age would negatively predict anxiety about aging (H3) and positively predict stereotypes toward the elderly (H4) and ageism, both directly (H5) and indirectly, via stereotypes about the elderly. Furthermore, anxiety about aging would positively predict stereotypes about the elderly (H6), which, in turn, would positively predict ageism (H7). Finally, we hypothesized the indirect effect of anxiety about aging on ageism (H8). We also hypothesized the mediating role of gender in the relationship among these variables.

## 2. Materials and Methods

### 2.1. Procedure of Recruitment and Participants

The participants were identified by snowball sampling that relied on a referral from initial participants, starting with university students, who used word-of-mouth to generate additional participants. The sampling and word-of-mouth process took place in the university from March to May 2018. Participation in the study was voluntary and anonymous, and participants were encouraged to answer as truthfully as possible. All data were collected with self-report questionnaires, which were administered individually in approximately 15–20 min.

Participants gave consent to participate in the study on the first page of the survey. The next page of the survey consisted of a presentation of four different instruments: Anxiety about Aging Scale, Fraboni Scale of Ageism, Aging Semantic Differential, and Facts on Aging Quiz (for a detailed description of the instruments, see the following section). A basic demographic questionnaire was completed on the final page of the survey, collecting information regarding each participant’s age and gender.

The study protocol was conducted according to APA and University Federico II ethical standards. In accordance with the provisions of Italian law, there being no treatment of persons, no authorization was required from the ethics committee, but it was only necessary to follow the rules proposed by it (see link at: http://www.comitatoeticofedericoii.it). The study conformed to the ethical principles of the 1995 Helsinki Declaration.

The convenience sample consisted of 886 Italian respondents (64.8% females, 35.2% males), aged from 18 to 65 years (M = 35.82, SD = 14.23), with the following distribution among age groups: 18–25 = 39.8%; 26–35 = 8.1%; 36–55 = 42.6%; 56–65 = 9.4%.

### 2.2. Instruments

*Fraboni Scale of Ageism*. The Italian version of the Fraboni Scale of Ageism (FSA) [27,29] consists of 19 statements arranged in a Likert format, ranging from 1 (strongly disagree) to 4 (strongly agree). It is designed to assess both cognitive and affective components of ageism. The FSA is composed of three factors: separation and avoidance (six items; e.g., “It is best that old people live where they won’t bother anyone”), stereotypes and antilocution (eight items; e.g., “Many old people just live in the past”), affective attitudes and discrimination (five items; e.g., “The company of most old people is quite enjoyable”. Items of this dimension are ‘reverse keyed’). Higher scores indicate higher levels of ageism. In this study, internal consistency reliability of the scale is 0.76, (Cronbach’s α), for the subdimensions α ranges from 0.62 to 0.78.

*Aging Semantic Differential*. The Italian version of the Aging Semantic Differential (ASD) [49,50,51] measures the impact of stereotypes on respondents’ attitudes concerning older adults on 20 pairs of opposite adjectives, which referred to four dimensions: integrity (seven items; e.g., “optimistic/pessimistic”), acceptability (four items; e.g., “friendly/unfriendly”), instrumental (five items; e.g., “active/passive”), and autonomy (four items; e.g., “organized/disorganized”). All items were measured on a seven point semantic differential scale. Higher scores indicate higher levels of stereotypical views. In this study the Cronbach’s alpha of the scale is 0.89, and α of the subdimensions ranges from 0.73 to 0.81.

*Anxiety about Aging Scale*. The Italian version of the Anxiety about Aging Scale (AAS) [47,51] consists of an 18 item self-report questionnaire that measures overall anxiety about aging on four subscales: (1) fear of old people (five items; e.g., “I enjoy being around old people”); (2) psychological concerns (four items; e.g., “I fear it will be very hard for me to find contentment in old age”); (3) physical appearance (four items; e.g., “I have never dreaded looking old”); (4) fear of loss (five items; e.g., “I fear when I am old all my friends will be gone”). All items were measured on a five point Likert scale, anchored at “strongly agree” and “strongly disagree”. Higher scores indicate higher levels of anxiety about growing old. In the current study, the Cronbach’s α of the scale is 0.78, and α of the subdimensions ranges from 0.65 to 0.78.

*Facts on Aging Quiz*. The Italian version of the Palmore Facts on Aging Quiz (FAQ) [34,51] is a measure of misconceptions concerning aging (e.g., “Physical strength declines in old age”). The FAQ consists of 25 true–false items. Correct items were summed so that higher scores indicated greater knowledge about the aging process.

### 2.3. Statistical Analysis

Next, survey data were entered into SPSS 22.0 databases [52] and Lisrel 8.54 software [53].

Cronbach’s alpha was used to calculate the reliability of the scales. For the psychological scale, an internal consistency should be greater than 0.70, even if an alpha between 0.60 and 0.69 would be considered acceptable [54].

Descriptive statistics were used to analyze the characteristics of the respondents and the study variables. To determine the relationships between all the variables, Pearson’s correlation coefficient was used (*p* < 0.05). Structural relationships were tested using Structural Equation Modeling (SEM). To assess the goodness-of-fit of the model, we used, as indicated, chi-squared distribution and the degrees of freedom (χ^2^/df ≤ 3), Standardized Root Mean Square Residual (SRMR ≤ 0.09), comparative fit index (CFI > 0.90), and non-normed fit index (NNFI > 0.90). If the results of the Root Mean Square Error of Approximation (RMSEA) are ≤ 0.05, they are considered to be good, and they are considered reasonable if they are ≤ 0.09. Evaluating multiple fit indices simultaneously is recommended because the different indices assess different aspects of goodness-of-fit [55,56,57]. Satisfactory models should show consistently good-fitting results on many different indices.

We tested factor invariance through several steps, as recommended by researchers in the field [57].

## 3. Results

### 3.1. Means, Standard Deviations, and Correlation Analysis

Means, standard deviations, and correlations are shown in Table 1.

### 3.2. Testing of the Hypothesized Conceptual Model and Invariance for Gender

**Figure 2:**
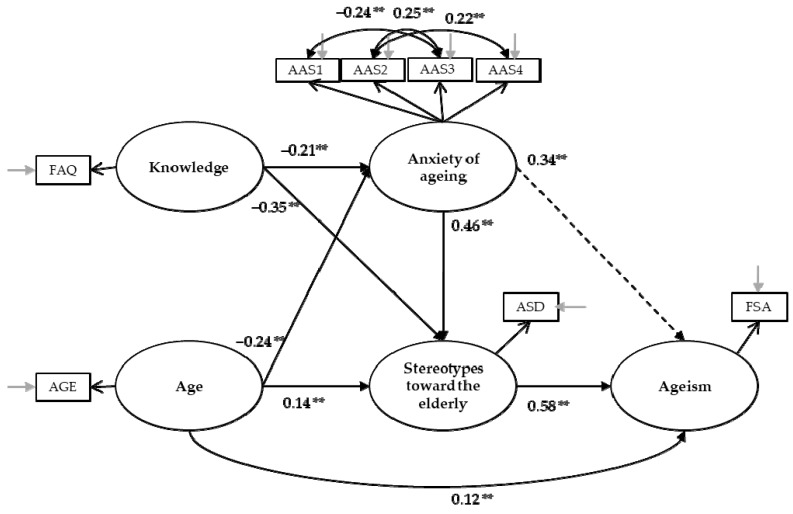
Structural equation model with standardized coefficients estimates (** *p* < 0.001).

We used structural equation modeling to test the structural relationships. The hypothesized model for predicting ageism was tested (Figure 2), and the results confirmed our model, with acceptable fit between the theoretical and the empirical models: χ^2^(df) = 23.46(10), *p* = n.s.; χ^2^/df = 2.35; CFI = 0.84; NFI = 0.83; RMSEA = 0.04 [0.02, 0.06]; SRMR = 0.087; GFI =1.00; AGFI = 1.00. As hypothesized, anxiety is negatively predicted by knowledge (H1; β = −0.21) and age (H3; β = −0.24). Stereotypes are predicted negatively by knowledge (H2; β = −0.35), and positively by age (H4; β = 0.14) and anxiety (H6; β = 0.46). Finally, ageism is positively predicted by stereotypes (H7; β = 0.58) and age (H5; β = 0.12). The indirect effect of anxiety about growing old is also significant on ageism (H8; β = 0.34).

To be able to verify invariance for gender, the model was tested separately for men and for women. The indices of fit of the tested model, for men only, were not satisfactory [χ^2^(df) = 26.40(10), *p* = n.s.; χ^2^/df = 2.64; CFI = 0.58; NFI = 0.58; RMSEA = 0.07 [0.04, 0.11]; SRMR = 0.13; GFI =1.00; AGFI = 1.00]. In contrast, those for the tested model with women only were satisfactory and even better than the general model: χ^2^(df) = 15.24(10), *p* = n.s.; χ^2^/df = 1.52; CFI = 0.93; NFI = 0.92; RMSEA = 0.030 [0.00, 0.059]; SRMR = 0.059; GFI =1.00; AGFI = 1.00]. For the sample of women, as well as for the general sample, anxiety is negatively predicted by knowledge (H1; β = −0.20) and age (H3; β = −0.17). Stereotypes are predicted negatively by knowledge (H2; β = −0.35) and positively by age (H4; β = 0.23) and anxiety (H6; β = 0.54). Ageism is positively predicted by stereotypes (H7; β = 0.59) and age (H5; β = 0.06). Finally, the indirect effect of anxiety about growing old is also significant on ageism (H8; β = 0.33).

## 4. Discussion

Growing awareness of the elderly, caused by an exponential growth in the aging population, has led scholars to investigate the factors that, in different ways, affect discriminatory and stereotypical attitudes toward the elderly. Many of the results obtained, however, do not point in the same direction, whether due to methodological issues [23] or because we are dealing with a phenomenon that has many notable cultural influences [25]. This requires researchers to proceed with further research using validated instruments, which are helpful in accurately capturing contextual specifics. The large number of studies conducted until now has, in any case, provided a picture of the relationships between all the variables involved, but has utilized a fragmentary approach. Furthermore, the current study has taken as its goal that of simultaneously testing, for the first time, some of the variables principally involved in this process. In light of the empirical evidence, therefore, we have outlined a model that considers the relationships between knowledge of aging, age, stereotypical attitudes toward the elderly, anxiety about aging, and ageism. The tested model has acceptable indices of adaptation. The hypotheses we formulated regarding the relationships were all confirmed. From the model, it emerges that age is an important factor insofar as, on the one hand, it negatively impacts anxiety about aging and, on the other, it positively impacts stereotypical attitudes toward the elderly and ageism, even if this is so with low predictive power, most likely due to an imperfect balance among the age groups in the sample. This indicates that growing older comes with diminished anxiety about aging, but also increases stereotypes and prejudices about aging. Young people, therefore, show a greater preoccupation with the transformations they imagine come with advanced age, which is consistent with the study conducted by Lasher and Faulkender [47]. Worries about the outcomes of the aging process reflect personal fears about aging and are probably related to an inner desire to satisfy social ideals of youth, typical of western societies, which promote an anti-aging culture [58]. The fact of a positive relation between age and stereotypes, and prejudices about aging, is in line with what has already been shown by the work of Hellbusch and colleagues [32], in which they surveyed the tendency of elderly people to have a more prejudiced attitude toward their own age group. This data may seem contradictory to that of the studies that have emphasized the existence of more antagonistic stereotypical attitudes toward the elderly in young people [59,60], but actually, the studies that have shown the existence of such a negative correlation were conducted on university students—a very limited age group [30,31] compared to that of the current study. Looking at age in relation to stereotypes and prejudices toward the elderly, using a structural equation model on a sample that comprises an age range from young people to adults on the threshold of old age, has allowed us to better capture the role of this variable. Furthermore, this data is consistent with that found in studies that examined the existence of prevalent stereotypical attitudes and prejudices in elderly adults [61]. Thus, from the results, the centrality of the role played by knowledge about aging emerges. Knowledge negatively impacts both anxiety about aging and stereotypical attitudes toward the elderly. Furthermore, it confirms that greater knowledge about aging is predictive both of lessened anxiety about aging [46] and of more positive stereotypical attitudes toward the elderly [41]. Anxiety about aging is, then, a predictor of stereotypical attitudes toward the elderly, which, in turn, positively predict ageism both in a direct way and as a mediator in the relation between anxiety about aging and ageism. The relationships between these variables is therefore clarified, and the role of knowledge about aging emerges more clearly, showing that it could lead to a more positive attitude and to less prejudice.

Furthermore, by aiming to verify the mediating role of gender in the relationships between the tested variables, it was possible to ascertain that these relationships are specific to women, which makes age a less powerful predictor for anxiety about aging, as well as for ageism, the standardized score for which, while meaningful, becomes nearly irrelevant. Finally, we emphasize the greater predictive power of anxiety about aging, relative to stereotypes.

This study has had the advantage of offering a more complete picture of a complex process—that of ageism; at the same time, we emphasize that the study has various limits, the first among which is the indices of adaptation in the model of the data. Some of the indices presented have values near the limit, which can be explained in light of the model’s lack of applicability to men, and this demonstrates the need for further investigation. Secondly, the sample is not representative of the Italian population and, therefore, repeated studies are encouraged in order to verify the stability of the model. Lastly, this has been a cross-sectional study that used a self-report questionnaire, and, in the future, it would be wise to use longitudinal studies and both quantitative and qualitative methods.

## 5. Conclusions

In recent decades, the study of ageism has increased due to the growing elderly population. Ageism is quite different from other forms of prejudice because it represents bias and discrimination by members of one group against members of a second group which the first group will one day join. In fact, categorization of an individual by age is not static, and changes over the course of the life cycle. This makes it different from discrimination based on race and gender, which remain consistent categories [62]. Furthermore, all individuals are destined to become old, unless death arrives before they can experience old age [63]. This peculiarity introduces new elements to the process of defining prejudice, first among which is anxiety for one’s own future aging. Young adults, anxious about their future, attribute to older people the negative stereotypes that they fear will describe their own futures [37]. According to Levy’s stereotype embodiment theory, stereotypes about older adults are internalized during childhood and, often unconsciously, they produce attitudes, expectations, and perceptions regarding the aging process [18]. The stereotypes that are internalized during youth, which intensify over time, can be contrasted with an accurate understanding of the various phases of the life cycle [44]. This suggests the importance of preventive programs that expand knowledge about the aging process, in order to reduce anxiety about aging and to promote a less-stereotyped image of the elderly. These programs should take into account gender differences in the socially-constructed process of aging, as well as in anxiety about aging and stereotypes about old age. Furthermore, there should be early intervention since, as McGuire and Mefford [64] say, it is easier to learn than to re-learn. From very young ages and for all educational levels, there should be activities aimed at spreading awareness of different types of characteristics in the aging process. Making an intervention among the youngest populations will guarantee to all of society’s future generations that they are no longer gripped by anxiety about what the future holds for them. Other interventions can be programmed for the age group which is no longer in its formative stages and is approaching old age. In members of this age group, it would be helpful to contrast the effects of negative, internalized stereotypes; furthermore, beyond offering accurate information, it will also be necessary to address those individual characteristics that are fundamental to a successful outcome for the aging process, such as self-esteem [65], self-efficacy [66], and locus of control [67,68]. Just by developing these capacities, people will adopt preventive behaviors, seek medical treatment, and no longer believe in negative stereotypes about inevitable declines in health due to aging [69]. In this way, it will be possible to avoid the dramatic effects of the internalization of stereotypes, with positive effects on the physical [70] and mental health of the elderly [71,72,73].

## Figures and Tables

**Table 1:** Correlations between the variables included in the study (** *p* < 0.01).

	M (SD)	AAS	FSA	ASD	FAQ
AAS_Anxiety about Aging Scale	2.62 (0.50)	1			
FSA_Fraboni Scale of Ageism	2.10 (0.35)	0.36 **	1		
ASD_Aging Semantic Differential	3.98 (0.79)	0.35 **	0.45 **	1	
FAQ_Facts on Aging Quiz	13.44 (2.74)	−0.24 **	−0.29 **	−0.31 **	1
Age	35.82 (14.23)	−0.16 **	0.14 **	0.02	0.04

SD: Standard Deviation; AAS: Anxiety about Aging Scale; FSA: Fraboni Scale of Ageism; ASD: Aging Semantic Differential; FAQ: Facts on Aging Quiz.
